# Effect of women’s fertility and sexual development on epigenetic clock: Mendelian randomization study

**DOI:** 10.1186/s13148-023-01572-z

**Published:** 2023-09-28

**Authors:** Boxin Zhang, Qizhi Yuan, Yining Luan, Jian Xia

**Affiliations:** 1 Department of Neurology, Xiangya Hospital, Central South University, 87 Xiangya Road of Kaifu District, Changsha, 410008 China; 2 Hunan Clinical Research Center for Cerebrovascular Disease, Changsha, China; 3 National Clinical Research Center for Geriatric Disorders, Xiangya Hospital, Central South University, Changsha, Hunan China

**Keywords:** AAM, AFS, AFB, ANM, Epigenetic clock, Mendelian randomization, Potential causal association

## Abstract

**Background and objectives:**

In observational studies, women’s fertility and sexual development traits may have implications for DNA methylation patterns, and pregnancy-related risk factors can also affect maternal DNA methylation patterns. The aim of our study is to disentangle any potential causal associations between women’s fertility and sexual development traits and epigenetic clocks, as well as to search for probable mediators by using the Mendelian randomization (MR) method.

**Methods:**

Instrumental variables for exposures, mediators, and outcomes were adopted from genome-wide association studies data of European ancestry individuals. The potential causal relationship between women’s fertility and sexual development traits and four epigenetic clocks were evaluated by inverse variance weighted method and verified by other two methods. Furthermore, we employed multivariable MR (MVMR) adjusting for hypertension, hyperglycemia, BMI changes, and insomnia. Then, combining the MVMR results and previous research, we performed two-step MR to explore the mediating effects of BMI, AFS, and AFB. Multiple sensitivity analyses were further performed to verify the robustness of our findings.

**Results:**

Leveraging two-sample MR analysis, we observed statistically significant associations between earlier age at first birth (AFB) with a higher HannumAge, PhenoAge and GrimAge acceleration(*β* = − 0.429, 95% CI [− 0.781 to − 0.077], *p* = 0.017 for HannumAge; *β* = − 0.571, 95% CI [− 1.006 to − 0.136], *p* = 0.010 for PhenoAge, and *β* = − 1.136, 95% CI [− 1.508 to − 0.765], *p* = 2.03E−09 for GrimAge respectively) and age at first sexual intercourse (AFS) with a higher HannumAge and GrimAge acceleration(*β* = − 0.175, 95% CI [− 0.336 to − 0.014], *p* = 0.033 for HannumAge; *β* = − 0.210, 95% CI [− 0.350 to − 0.070], *p* = 0.003 for GrimAge, respectively). Further analyses indicated that BMI, AFB and AFS played mediator roles in the path from women’s fertility and sexual development traits to epigenetic aging.

**Conclusions:**

Our study suggested that AFS and AFB are associated with epigenetic aging. These findings may prove valuable in informing the development of prevention strategies and interventions targeted towards women’s fertility and sexual development experiences and their relationship with epigenetic aging-related diseases.

**Supplementary Information:**

The online version contains supplementary material available at 10.1186/s13148-023-01572-z.

## Introduction

DNA methylation (the methylation of cytosine carbon 5 at cytosine phosphate guanine (CpG) dinucleotides) is a stable, yet reversible epigenetic modification. Biological age is loosely defined and strongly associated with tissue and organismal functional decline, age-related health conditions, morbidity, and mortality. Based on dynamic changes in epigenetic processes along the life-course, there have been developed multiple epigenetic clocks that are highly predictive of biological age [1].

The first generation of epigenetic clocks including HannumAge [2] and HorvathAge [3]. The HannumAge, previously denoted as ‘extrinsic’ epigenetic age acceleration (EEAA) in earlier research, can be viewed as a biomarker indicative of immune system aging. This marker explicitly encompasses elements of immune system decline, including age-related variations in blood cell counts, and it is associated with lifestyle and health-related traits [2], and HorvathAge, previously referred to in existing literature as ‘intrinsic’ epigenetic age acceleration (IEAA), can be understood as an indicator of cell-intrinsic aging, a concept that remains consistent across various tissue types [3]. The second generation of epigenetic clocks including PhenoAge [4] and GrimAge [5]. Among these, PhenoAge integrated data from CpGs associated with mortality and related clinical biomarkers [4]. Horvath et al. defined GrimAge by using CpGs associated with smoking history and disease-related and health-related proteins [5]. The first generation of clocks primarily relied on CpG sites in blood or tissues, along with some related variables, these two clocks are more predictive of chronological age than other clocks. In contrast, the second generation of clocks incorporate a larger number of CpG sites and also consider clinical biomarkers associated with mortality and disease. This advancement enables the second generation of clocks to more accurately predict individuals’ age-related disease risks and mortality risks. Besides, DNAm GrimAge outperformed the other three epigenetic clocks in its associations with disease data and associated clinical traits [6]. These epigenetic clocks have the potential to shed light on enduring questions across various fields, notably the fundamental inquiry: What is our biological age, and what factors contribute to the aging process.

Women’s fertility and sexual development traits (including age at menarche (AAM), age at natural menopause (ANM), age at first sexual intercourse (AFS), age at first birth (AFB) and number of sexual partners (NSP) have important implications for ageing diseases and lifespan [7, 8]. Clinical research findings suggest that Hannum age and Horvath age are positively correlated with age at first live birth and age at menarche [9]. However, there is still a lack of research on the impact of other women’s fertility and sexual development traits, such as AFS and ANM, on epigenetic age. And previous clinical studies have not encompassed the use of the latest generation of epigenetic clocks. Therefore, this study aims to extensively investigate the relationship between women’s fertility and sexual development traits and two generations of epigenetic clocks.

Furthermore, our understanding of the mechanisms by which women’s fertility and sexual development traits impact epigenetic age is still limited. Currently, there is a lack of detailed research specifically addressing this relationship. Hormonal regulation, inflammation and stress response, as well as environmental and lifestyle factors, are all potential contributors to the regulation of epigenetic clocks by women’s fertility and sexual development traits [10–12]. Previous research has shown that maternal DNA methylation patterns can be affected by risk factors related to pregnancy [13, 14]. This implies certain adverse changes that can occur during pregnancy, such as hypertension, hyperglycemia, BMI changes, and insomnia, could be mediators or confounders of the associations we observed. In this study, we choose these four unfavorable changes associated with women’s fertility and sexual development traits as potential mechanisms to investigate. In addition, previous studies have suggested that women’s reproductive factors are genetically correlated and causally related, and later AAM can lead to later AFS and AFB [15]. Therefore, we also hypothesize that AFS and AFB themselves may serve as mediating factors through which women’s traits affect epigenetic clocks.

Mendelian randomization (MR) refers to a group of genetic methods. It uses single nucleotide polymorphisms (SNPs) as instrumental variables (IVs) to test whether an exposure causes an outcome. Since alleles are randomly allocated during the process of gamete formation, comparing to observational studies, MR can effectively overcome the biases and avoid reverse causality [16]. Among them, two-sample MR is a popular method for potential causal inference that leverages genetic associations between exposures and outcomes from different datasets. And multivariable MR (MVMR) extends this approach by adjusting for multiple exposures and confounders simultaneously, allowing researchers to investigate the independent potential causal effects of multiple exposures on outcomes. Apart from those, two-step MR is a powerful method for investigating potential mediating factors in potential causal pathways. By first examining the potential causal relationship between exposure and mediator, and then between mediator and outcome, this approach allows researchers to test whether the effect of the exposure on the outcome is mediated by the mediator.

In this study, we performed two-sample univariable and multivariable MR to explore (i) the effect of women’s fertility and sexual development traits on epigenetic clocks, (ii) the independent effects of each women’s fertility or sexual development trait on epigenetic clocks after adjusting for confounders including insomnia, BMI, fasting glucose and hypertension. At last, we also performed two-step MR for mediation analysis to find the mediators of the potential causalities we found.

## Materials and methods

### Study design

In this study, we performed two-sample MR and MVMR analyses to clarify whether women’s reproductive traits (AFB, AFS, AAM, ANM and NSP) have potential causal effects on epigenetic clocks independent of BMI, hypertension, hyperglycemia and insomnia.

To ensure the validity of our MR analysis, we needed to confirm the fulfillment of three crucial assumptions. Firstly, we required that the instrumental variables (IVs) used in the analysis were strongly associated with the exposure variable under investigation. Secondly, the IVs needed to be independent of any other potential confounding factors that may affect the relationship between the exposure and outcome variables. Lastly, we assumed that the IVs would only affect the outcome variable through the exposure variable, and not via any other pathways [17]. MVMR assumes that genetic variants are associated with at least one of the exposures, while the remaining assumptions are similar to those of two-sample MR [18].

**Fig. 1:**
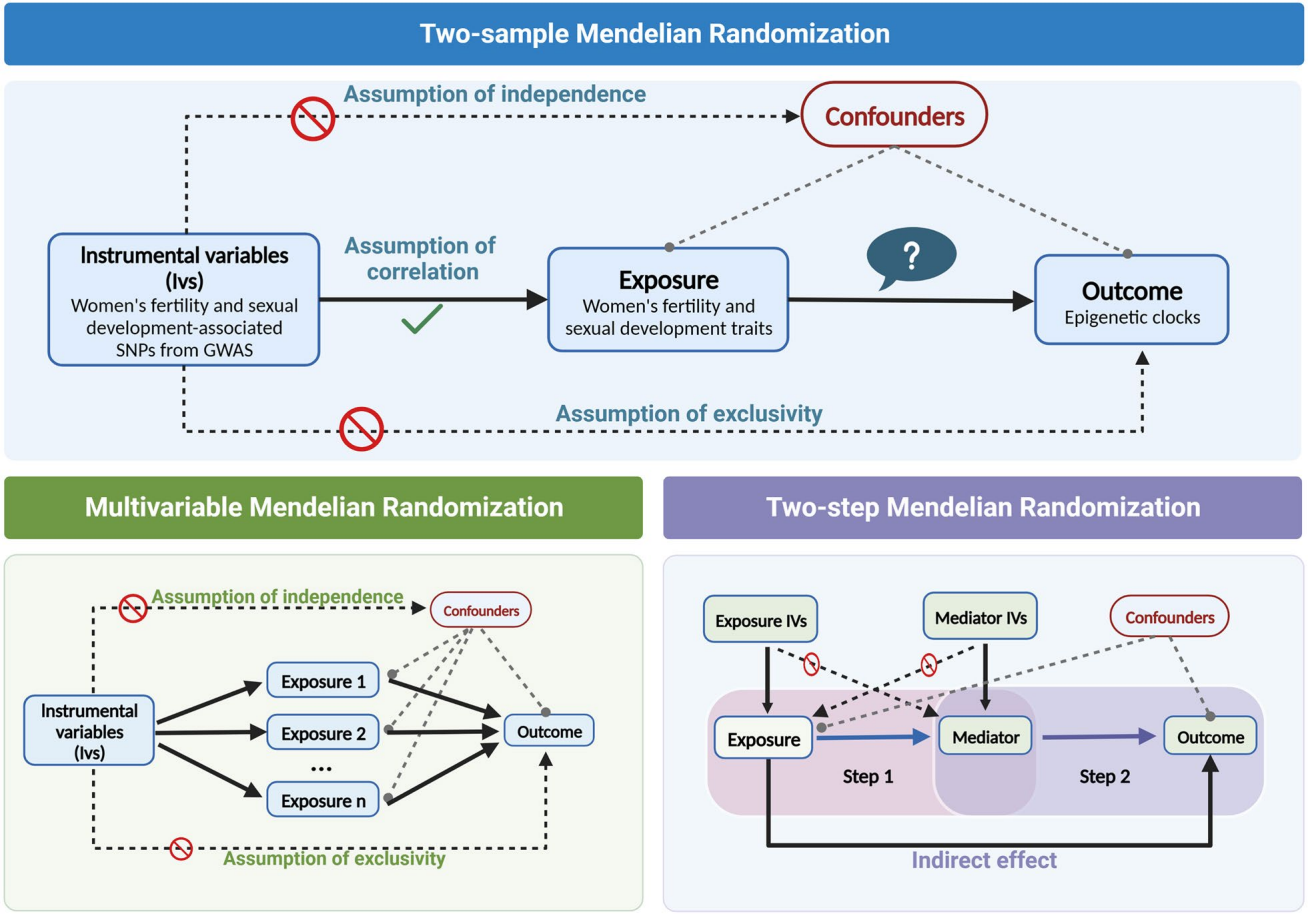
Assumptions and study design of the MR study of the associations between five women’s reproductive traits and four epigenetic clocks

We first investigated the potential causal relationship between female fertility or sexual development traits and epigenetic aging by using two-sample MR with large-scale GWAS data. Second, to estimate the direct impact of reproductive factors on the outcomes while controlling for the effects of insomnia, BMI, fasting glucose, and hypertension, we utilized MVMR models that integrated GWAS summary statistics and additional genetic variants related to these confounding factors. Third, we explored the role of BMI, AFB and AFS (mediated mechanism) playing in the relationships of AFB, AFS and AAM on certain epigenetic clocks by two-step MR and calculated each mediation effect size (Fig. 1).

### Participants

We searched the literature, in the OpenGWAS and GWAS Catalog, for genetic instruments associated with AAM, ANM, AFS, AFB, NSP, BMI, insomnia, hypertension, fasting glucose and epigenetic clocks in populations of European ancestry. Additional file 1: Table S16 shows a summary of the GWAS use in exposures and confounders.

#### Genetic associations for exposures

Genetic variants of age at menarche were obtained from the combined analysis of up to 182,416 women from 57 studies, and women with a reported age at menarche as < 9 years or > 17 years were excluded from the analysis [19]. And we evaluated genetic instruments of age at natural menopause from the European ancestry meta-analysis in up to 69,360 women of European ancestry. For this meta-analysis, women with ANM 40–60 years were included and women with non-natural menopause were excluded [20]. The summary-level data for age at first birth and age at first sexual intercourse were obtained from a meta study consisting of 36 cohorts. For AFS, Participants who reported their AFS lower than 12 were excluded [21]. For lifetime number of sexual partners measurement instruments, we used a published large-scale GWAS of the UKB with up to 370,711 participants [22].

#### Genetic associations for outcomes

From this study, we obtained summary genetic association estimates for four epigenetic clock acceleration measures of: PhenoAge, GrimAge, HannumAge, and HorvathAge from a meta-analysis of European ancestry of 34,710 participants from 28 cohorts [23]. There was no overlap between exposure and outcome samples since exposures and outcomes data were obtained from different consortia.

#### Genetic associations for mediators

For insomnia, we acquired genetic instruments of insomnia complaints using the UK Biobank genome-wide association analysis including 113,006 individuals [24]. Besides, SNPs associated with BMI were acquired from a meta-analysis of genome-wide association studies in in up to 681,275 individuals of European ancestry [25]. Genome-wide association study data for hypertension were obtained from UK Biobank (www.ukbiobank.ac.uk) in up to 408,652 participants. For fasting glucose instruments, we used a published genome-wide association analysis in up to 200,622 participants without diabetes (70% European ancestry) [26].

### Statistical analysis

#### Two-sample MR

Statistical analysis was conducted using R version 4.2.1 with the ‘Two-Sample MR’ and ‘MR-PRESSO’ packages [27, 28]. We obtained SNPs from corresponding genome-wide association studies (GWASs) for each exposure trait, with a genome-wide significance level set at *p* < 5 × 10^−8^ [5]. To ensure independence, we pruned the selected instrumental variables (IVs) using a clumping *r*^2^ cutoff of 0.05 on a 1 Mb window before conducting MR analysis. We also harmonized each pair of the exposure and outcome datasets, removed palindromic single nucleotide variants (SNVs) with intermediate allele frequencies, and verified that the selected IVs were not associated with confounding factors using PhenoScanner (www.phenoscanner.medschl.cam.ac.uk/) [29]. The SNPs that were removed and the IVs that were ultimately used can be found in the Additional file 1.

We utilized the inverse variance–weighted (IVW) MR method as the primary analysis to calculate the overall estimate. Although the weighted median (WM) and MR-Egger methods were less statistically powerful, they were more robust to horizontal pleiotropy [30]. Therefore, we employed the MR-weighted median and MR-Egger methods as supplementary analyses to confirm the stability of the results, and we used the intercept of the MR-Egger method to identify any significant evidence of horizontal pleiotropy. Additionally, we used the MR-PRESSO global test to detect any potential horizontal pleiotropy and performed the MR-PRESSO outlier test to correct for any horizontal pleiotropy via outlier removal [28]. Furthermore, we conducted leave-one-out analyses to assess if a particular SNP significantly influenced the MR estimates [31]. Finally, Cochran’s Q tests were employed to check for heterogeneity across the instrumental variables (IVs).

#### Multivariable MR

To account for the potential confounding or mediating roles of insomnia, BMI, glucose, and hypertension in the pathway from female fertility or sexual development traits to epigenetic clocks, we conducted multivariable Mendelian randomization (MVMR) analysis [32]. We used an extension of the IVW MR method, known as MVMR-IVW, and selected random effects or fixed effects based on heterogeneity, as described in the two-sample MR. Specifically, we included insomnia, BMI, glucose, and hypertension as covariates in the MVMR analysis to examine the direct effect of female fertility or sexual development traits on epigenetic clocks while controlling for these potential confounders. Among women, there was a robust genetic correlation observed between AFB and AFS [33]. We conducted a multivariable Mendelian randomization (MVMR) analysis that incorporated both AFB and AFS as exposures to consider the genetic correlation and examine the individual effects of each exposure on epigenetic clocks.

#### Exploration on the mediator

The MVMR results suggested that the confounding factor BMI had an independent potential causal relationship with the outcome, indicating its potential role as a mediator. In conjunction with the results of MVMR and previous study, we further conducted two-sample tests on the exposure and possible mediating factors, which included BMI, AFB, and AFS. Additionally, the relationship of the mediator BMI to the outcomes were examined. For the group with positive results of both exposure-outcome, exposure-mediating factor and mediation-outcome, we further performed two-step MR To explore the mediating effect. We further confirmed the significance of this mediation effect using the product of coefficients method.

## Result

### Two-sample Mendelian randomization of female fertility and sexual development traits on epigenetic clocks

**Fig. 2:**
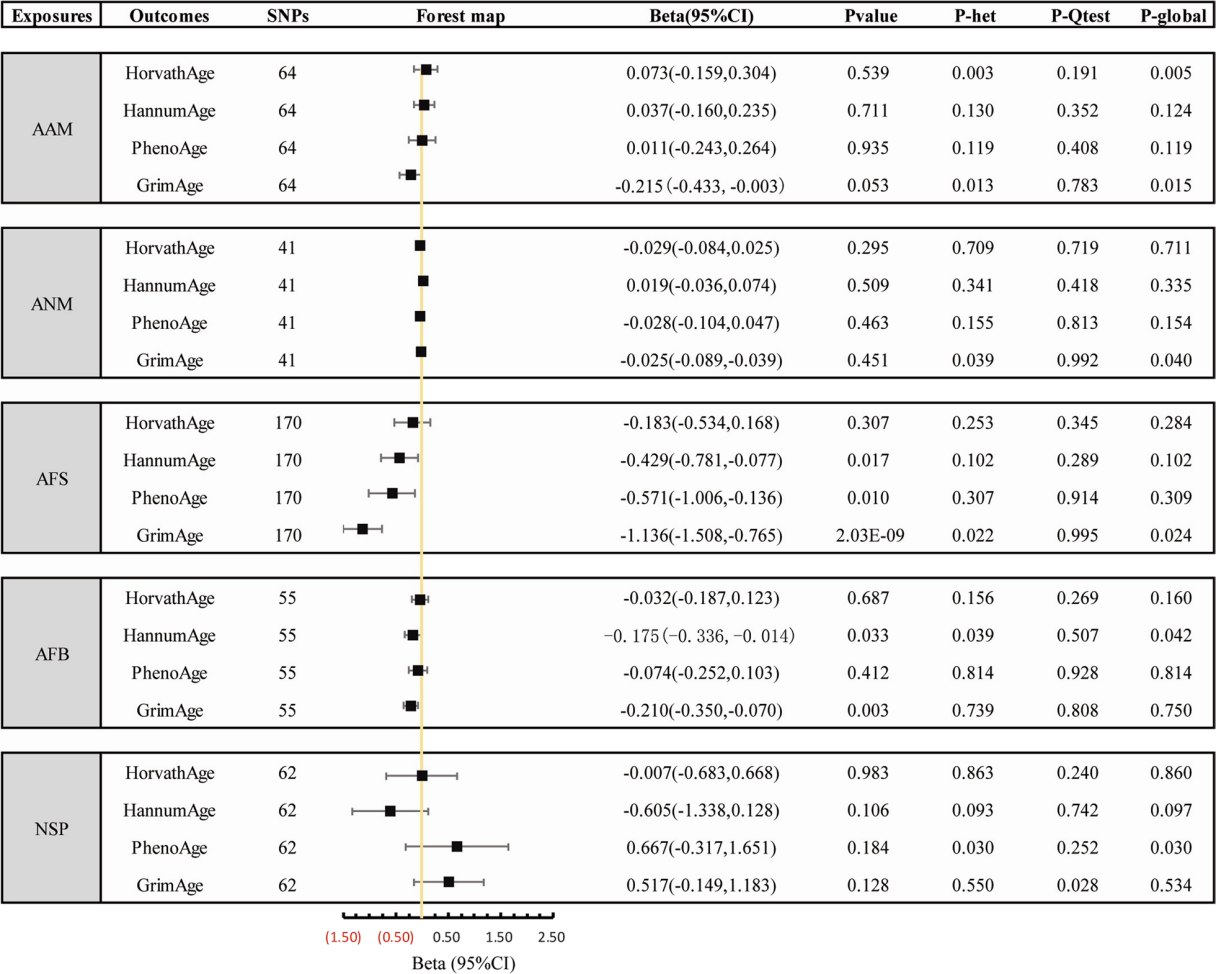
Two-sample Mendelian randomization analysis of the potential causal effects of five women’s reproductive traits on four epigenetic clocks. *AAM* Age at menarche; *ANM* Age at natural menopause; *AFS* Age at first sexual intercourse; *AFB* Age at first birth; *NSP* Number of sexual partners; *SNP* Single-nucleotide polymorphism

The forest plots (Fig. 2) display the association between genetically elevated female fertility and sexual development traits and epigenetic clocks. IVW analysis showed that there were negative potential causal associations between AFS, AFB and epigenetic age acceleration.

**Fig. 3:**
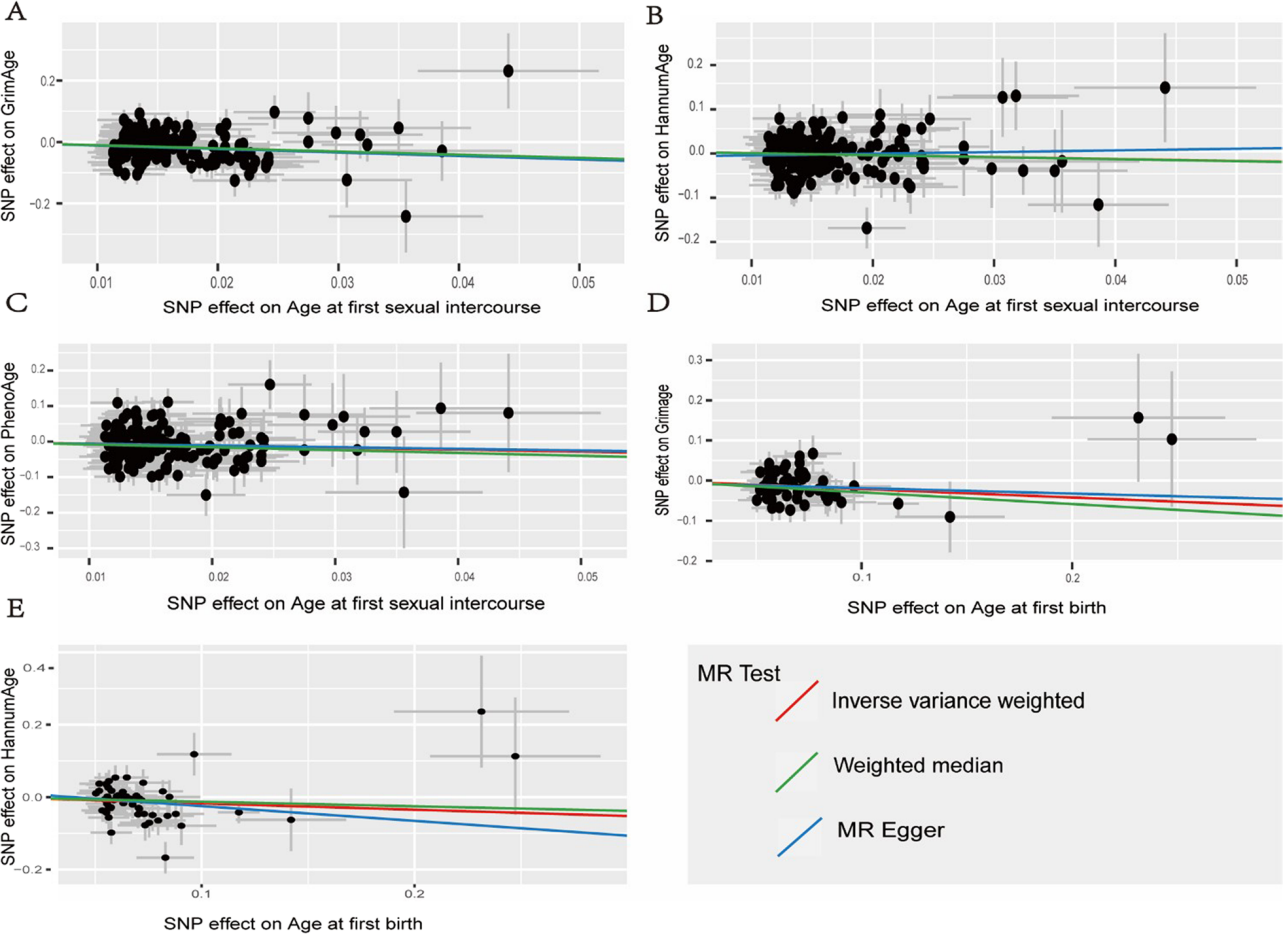
Scatter plots for five positive results in the two-sample Mendelian randomization analyses. The error bars indicate standard error. **A** AFS on GrimAge. **B** AFS on HannumAge. **C** AFS on PhenoAge. **D** AFB on GrimAge. **E** AFB on HannumAge

After clumping at *r*^2^ < 0.001, we identified 216 SNPs to proxy for an SD increase in AFS at *P* ≤ 5 × 10^−8^, of which 4 genes were missing in the outcome data set. Then 42 SNPs were excluded because they were palindromic with intermediate allele frequencies (Additional file 1: Table S2), resulting in 67 independent genetic instruments (Additional file 1: Table S2). The F statistics for individual SNPs were all larger than 10 (Additional file 1: Table S2), with a mean F statistic of 44.09, suggesting that weak instruments bias may not be substantial. IVW MR analysis with a random-effects model by 4 epigenetic clocks revealed that genetically predicted one SD increase in AFS could reduce the epigenetic age acceleration (*β* = − 0.429, 95% CI [− 0.781 to − 0.077], *p* = 0.017 for HannumAge; *β* = − 0.571, 95% CI [− 1.006 to − 0.136], *p* = 0.010 for PhenoAge, and *β* = − 1.136, 95% CI [− 1.508 to − 0.765], *p* = 2.03E−09 for GrimAge respectively). We observed similar associations with the weighted median method for AFS on PhenoAge and GrimAge (Beta-0.799, 95% CI [− 1.447 to − 0.151], *p* = 0.016 for PhenoAge, and Beta-1.032, 95% CI [− 1.553 to − 0.511], *p* = 1.02E−04 for GrimAge, respectively), but not with the MR-Egger method (Additional file 1: Table S1, Fig. 3).

We obtained 55 SNPs as genetic instruments for AFB (Additional file 1: Table S3) after excluding 10 SNPs that were missing in outcome data or palindromic. The F statistics for individual SNPs were all larger than 10 (Table S3), with a mean F statistic of 37.87. The random effect IVW analysis showed that higher AFB were correlated inversely with epigenetic age acceleration (*β* = − 0.175, 95% CI [− 0.336 to − 0.014], *p* = 0.033 for HannumAge; *β* = − 0.210, 95% CI [− 0.350 to − 0.070], *p* = 0.003 for GrimAge, respectively). Weighted median analysis also showed a negative potential causal association between AFB and GrimAge (*β* = − 0.292, 95% CI [− 0.506 to − 0.078], *p* = 0.007) (Additional file 1: Table S1, Fig. 3).

We observed no potential causal relationship between other female fertility and sexual development traits and epigenetic clocks from MR.

As for the sensitivity analyses, all of the *p* values from the Q statistic were more than 0.05, and the leave-one-out analysis showed no heterogeneity in their effects on outcomes. Unfortunately, parts of the MR-Egger intercept test and MR PRESSO global test showed heterogeneity in AFS and AFB effects on outcomes (Additional file 1: Tables S1, S2). Additional studies are needed to demonstrate a potential causal link between AFS, AFB and epigenetic clocks.

### MVMR analyses of each female fertility and sexual development traits on epigenetic clocks

**Fig. 4:**
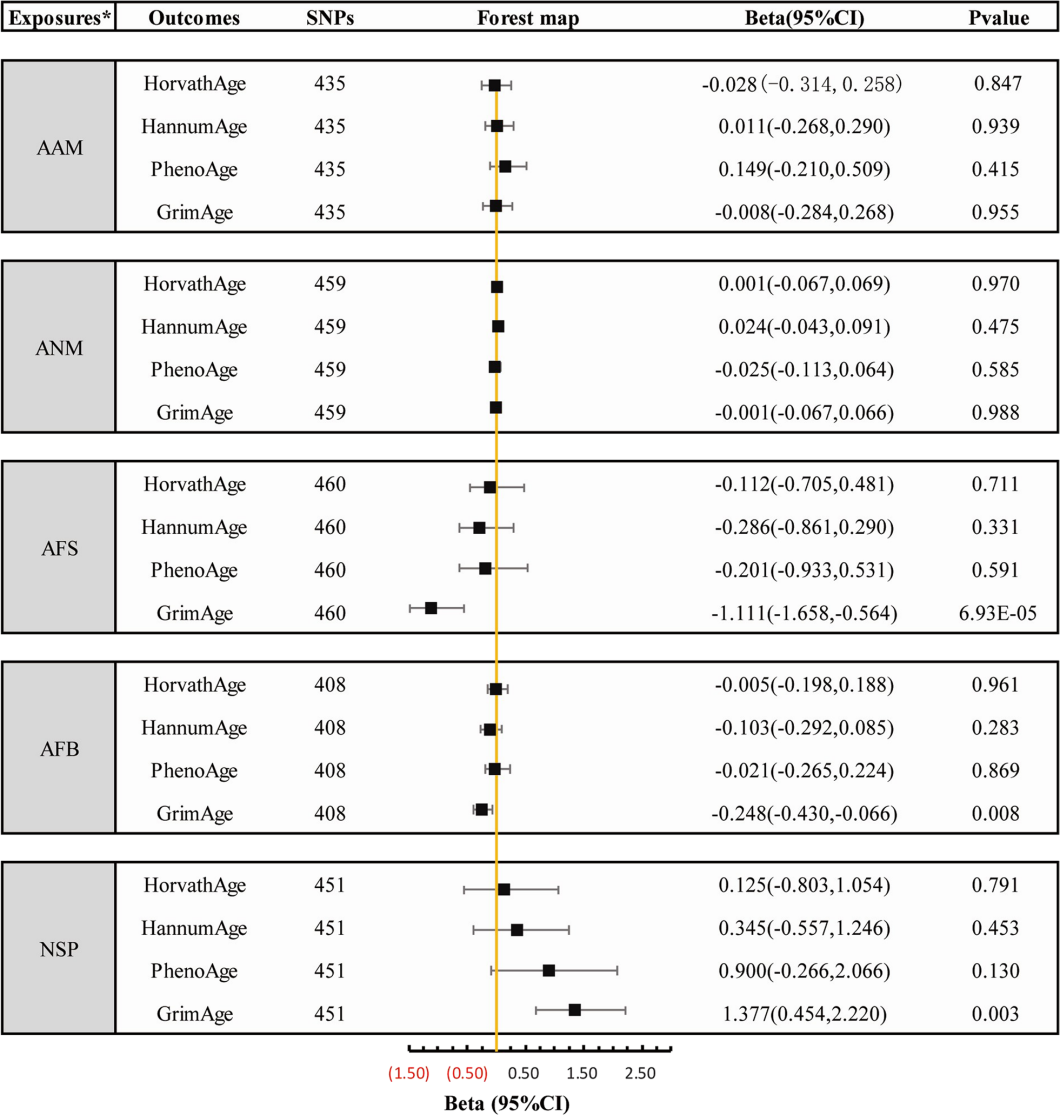
Multivariable Mendelian randomization analysis of the potential causal effects of five women’s reproductive traits on four epigenetic clocks controlled for insomnia, body mass index, fasting glucose and hypertension. *AAM* Age at menarche; *ANM* Age at natural menopause; *AFS* Age at first sexual intercourse; *AFB* Age at first birth; *NSP* Number of sexual partners; *SNP* Single-nucleotide polymorphism

We applied multivariable Mendelian randomization analyses for the assessment of potential causal relationships between each female fertility or sexual development trait and epigenetic clocks after controlling for insomnia, BMI, glucose and hypertension. The association between AFS and HannumAge or PhenoAge was no longer significant after controlling for the effect of insomnia, BMI, glucose and hypertension compared with results of two-sample MR (all *p* > 0.05,Fig. 4). Besides, there was strong evidence for a direct potential causal effect of AFS on GrimAge (*β* = − 1.111, 95% CI [− 1.658, − 0.564], *p* = 6.93E−05, Fig. 4). The results of the MVMR analysis revealed that the potential causal relationship between AFB and GrimAge remained stable after adjusting for these four confounders (*β* = − 0.248, 95% CI [− 0.430, − 0.066], *p* = 0.008), while the potential causal associations of AFB with Hannum attenuated to be not significant after adjusting for insomnia, BMI, glucose and hypertension (*p* > 0.05, Fig. 4). Interestingly, after adjusting for four confounding factors, the potential causal relationship between NSP and GrimAge became significant, which was different from the results of the Two-sample MR (*β* = 1.377, 95% CI [0.454, 2.220], *p* = 0.003, Fig. 4).

Interestingly, for AFB, AFS and epigenetic clocks, MVMR results suggested BMI as a possible mediator. Besides, the effect of BMI on epigenetic clock in MVMR remains significant (Additional file 1: Tables S3–S6).

### Results of MR analyses on the mediator

For traits that produce positive results in two sample MR analysis, we further investigated the association between exposures, possible mediators and outcomes by using a series of two sample MR analyses (Table 1). The results of these two-sample MR confirmed that BMI, AFB and AFS were all potential mediators. As shown in Table 1, potential causal associations with BMI were all negative for AFB, AFS and AAM (*β* = − 0.104, 95% CI [− 0.134 to − 0.073], *p* = 3.50E−11 for AFB; *β* = − 0.338, 95% CI [− 0.427 to − 0.249], *p* = 1.15E−13 for AFS, and *β* = − 0.156, 95% CI [− 0.222 to − 0.089] for AAM, respectively), *p* = 4.50E−06 for AAM). Interestingly, higher AAM was associated with higher AFS and higher AFB (*β* = 0.205, 95% CI [0.100 to 0.309], *p* = 1.19E−04 for AFB; *β* = 0.093, 95% CI [0.061 to 0.125], *p* = 1.18E−08 for AFS, respectively). Besides, higher AFS often predicted higher AFB (*β* = 2.046, 95% CI [1.901 to 2.191], *p* = 6.75E−169).

**Table 1:** Results of MR analysis for selected exposures on possible mediators and MR analysis for possible mediator on selected outcomes

Exposure	Outcome	*β* (95% CI)	*p*	SE
AFB	BMI	− 0.104 (− 0.134, − 0.073)	3.50E−11	0.016
AFS	AFB	2.046 (1.901, 2.191)	6.75E−169	7.39E−02
	BMI	− 0.338 (− 0.427, − 0.249)	1.15E−13	0.046
AAM	AFB	0.205 (0.100, 0.309)	1.19E−04	5.32E−02
	AFS	0.093 (0.061, 0.125)	1.18E−08	1.63E−02
	BMI	− 0.156 (− 0.222, − 0.089)	4.50E−06	3.39E−02
BMI	HannumAge	0.163 (− 0.021, 0.348)	0.083	9.41E−02
	PhenoAge	0.528 (0.290, 0.767)	1.37E−05	1.22E−01
	GrimAge	0.595 (0.409, 0.782)	3.66E−10	9.50E−02

IVW methods showed elevated BMI was potentially associated with two accelerated epigenetic clocks (*β* = 0.528, 95% CI [0.290 to 0.767], *p* = 1.37E−05 for PhenoAge; *β* = 0.595, 95% CI [0.409 to 0.782], *p* = 3.66E−10 for GrimAge, respectively, Table 1).

Next, we undertook two-step MR analyses to evaluate the mediating effects of mediators on the potential causal links we found in the first part. After evaluating the mediation hypotheses by using the product of coefficients method, we found BMI played mediated role The analysis results revealed that the effect of AFS was partially mediated by BMI for PhenoAge (proportion mediated, 31.17%, 95% CI 19.6–42.7%; *p* = 0.007), and GrimAge (proportion mediated, 17.7%, 95% CI 12.5–22.9%; *p* = 5.97E−04); and partially mediated by AFB for HannumAge (proportion mediated, 83.4%, 95% CI 48.0–118.9%; *p* = 0.018), and GrimAge (proportion mediated, 37.8%, 95% CI 24.5–51.2%; *p* = 0.005) (Table 2). Likewise, AFB played mediated role in the relationships of AAM and GrimAge (partial mediation:20.0% of total effect, mediation effect 95% CI 11.2–28.8%; *p* = 0.020, Table 2).

**Table 2:** Results of mediation analysis using two-step MR

Exposures	Mediators	Outcomes	Total effect	Direct effect A	Direct effect B	Mediation effect	*p*	Mediated proportion (%)
			Effect size (95% CI)	Effect size (95% CI)	Effect size (95% CI)	Effect size (95% CI)		Effect size (95% CI)
AFB	BMI	PhenoAge	− 0.074 (− 0.252, 0.103)	− 0.104 (− 0.134, − 0.073)	0.528 (0.290, 0.767)	− 0.055 (− 0.119, 0.009)	0.392	/
		GrimAge	− 0.210 (− 0.350, − 0.070)	− 0.104 (− 0.134, − 0.073)	0.595 (0.409, 0.782)	0.062 (0.005, 0.119)	0.274	29.5 (2.3,56.6)
	AFB	HannumAge	− 0.429 (− 0.781, − 0.077)	2.046 (1.901, 2.191)	− 0.175 (− 0.336, − 0.014)	0.358 (0.206, 0.510)	0.018	83.4 (48.0,118.9)
AFS		GrimAge	− 1.136 (− 1.508, − 0.765)	2.046 (1.901, 2.191)	− 0.210 (− 0.350, − 0.070)	0.430 (0.278, 0.582)	0.005	37.8 (24.5,51.2)
	BMI	PhenoAge	− 0.571 (− 1.006, − 0.136)	− 0.338 (− 0.427, − 0.249)	0.528 (0.290, 0.767)	0.178 (0.112, 0.244)	0.007	31.17 (19.6,42.7)
		GrimAge	− 1.136 (− 1.508, − 0.765)	− 0.338 (− 0.427, − 0.249)	0.595 (0.409, 0.782)	− 0.201 (− 0.260, − 0.142)	5.97E − 04	17.7 (12.5,22.9)
AAM	AFB	GrimAge	− 0.215 (− 0.433, − 0.003)	0.205 (1.106, 1.362)	− 0.210 (− 0.350, − 0.070)	0.043 (0.024, 0.062)	0.020	20 (11.2,28.8)
	AFS	GrimAge	− 0.215 (− 0.433, − 0.003)	0.093 (0.061, 0.125)	− 1.136 (− 1.508, − 0.765)	− 0.106 (− 0.321, 0.109)	0.623	/
	BMI	GrimAge	− 0.215 (− 0.433, − 0.003)	− 0.156 (0.801, 0.915)	0.595 (0.409, 0.782)	− 0.093 (− 0.150, − 0.036)	0.101	43.3 (16.7,69.8)

## Discussion

Using the MR approach, we investigated the potential causal associations between women’s fertility and sexual development traits and epigenetic clocks. Our findings suggest that earlier AFB, AFS and AAM were potentially associated with increased epigenetic aging, and these effects may be attributed to an increase in BMI or the interaction of women’s fertility and sexual development traits themselves. This indicates the presence of a window of opportunity to mitigate the adverse impact of female reproductive-related experiences on the risk of epigenetic outcomes.

### Potential causal associations of each women’s reproductive trait with epigenetic clocks

Previous research demonstrated that younger AFS may put girls at risk for unwanted teen pregnancy, sexually transmitted infections, and poor social, emotional, and physical health outcomes in adolescence and adulthood [34], which had been found could lead to premature biological aging [35]. Our findings were consistent with this prior study. Leveraging two-sample MR analysis, we found that genetically predicted increase in AFS could reduce the HannumAge, PhenoAge and GrimAge acceleration. Similarly, IVW analysis showed that higher AFB were correlated inversely with HannumAge and GrimAge acceleration. Of note, in our analysis, we did not observe any significant evidence of HorvathAge’s effect in several relationships. Among the four clocks, HorvathAge demonstrated the closest association with actual chronological age, implying its relative insensitivity to pregnancy-related factors [36], this may account for the absence of positive results related to HorvathAge in our study.

Given that women reproductive traits were complicated traits shaped by both genetic and environmental factors, we performed an MVMR to control for the effect of BMI, hypertension, FBG and insomnia. The results of MVMR suggest that the potential causal relationship between AFS and AFB on GrimAge remains stable after controlling for confounding factors, suggesting that these two exposures can independently affect GrimAge. It is worth noting that different clocks have been established based on age-related CpGs and algorithms, each with its own advantages in diverse conditions [37]. Our findings suggest that AFS and AFB may have a direct impact on the composite of biomarkers associated with GrimAge, potentially influencing the onset of diseases.

### BMI, AFB and AFS as mediators in AFS/AFB/AAM and epigenetic clocks

Subsequently, our study found that AFS and AFB were causally associated with an increase in BMI, which in turn may accelerate epigenetic aging. Our study revealed that BMI acted as a mediator that explained 31.17% of the relationship between AFS and PhenoAge, 17.7% of the relationship between AFS and GrimAge, 83.4% of the relationship between AFB and HannumAge, 37.8% of the relationship between AFB and GrimAge. Likewise, AFB played mediated role in the relationships of AAM and GrimAge (partial mediation:20.0% of total effect). According to previous findings, insulin resistance may be partly responsible for the effects of obesity on epigenetic aging [38]. In addition, higher BMI increased levels of several DNA methylation-based plasma markers, including DNAm PAI-1 and TIMP-1, which are associated with markers of inflammatory and metabolic conditions [5]. As second-generation DNA methylation clock, GrimAge is a significant predictor of mortality risk and can strongly predict the cognitive dysfunction, functional limitations and chronic conditions [39, 40]. In this case, avoidance of obesity offers the potential of reducing epigenetic aging related diseases of women especially with special reproductive experiences, more studies are needed to confirm these findings.

### Strengths and limitations

One of the key strengths of our study is the use of MR analysis, which helps to establish potential causal inference and overcome the issue of confounding in such studies. In addition, we conducted multiple sensitivity analyses that provided additional reassurance and suggested that the observed associations are likely to be genuine. These findings increase our confidence in the robustness and validity of our results, and suggest that the relationships between AFS/AFB, BMI, and epigenetic aging that we have identified may be potentially causal in nature.

The limitations of our research are as follows: First, the datasets used in our study were derived from the European population, which may limit the generalizability of our findings to populations outside of Europe. Secondly, although BMI is the most commonly used indicator for obesity, it does not incorporate information on lean body mass or abdominal obesity. Therefore, in future research, we need to conduct more detailed investigations on waist circumference (WC) and waist-hip ratio (WHR), which reflect abdominal obesity, as well as body fat percentage (BFP), which indicates overall obesity. Furthermore, the summary-level data for exposures were only available for women, while epigenetic clock traits were assessed in both men and women. Furthermore, it’s worth emphasizing that we have merely skimmed the surface regarding the intermediary factors we’ve identified. Hormones, inflammation, mental health conditions… There are still numerous intermediary factors awaiting thorough exploration. Last but not least, in this study, we limited our analysis to examining the associations between four classic hematologic epigenetic clocks and female reproductive factors. As the field of epigenetics continues to advance with the development of methylation arrays, sequencing technologies, and other relevant techniques, an increasing number of epigenetic clocks have been developed. Correspondingly, data from GWAS related to these markers have been accumulating and improving. Therefore, in future investigations, it would be valuable to consider broadening the scope by including a wider array of epigenetic clocks to comprehensively investigate their potential connections with reproductive health.

## Conclusion

In conclusion, our study supports the potential causal association between women fertility and sexual development traits on epigenetic clock, in which BMI, AFB and AFS may play mediator roles in these potential causal relationships. Our findings advance research on epigenetic aging by demonstrating that both AFS and AFB could accelerate biological aging measured with epigenetic clocks by mediating effect of mediators as well as independent effect.

### Supplementary Information

**Additional file 1 Table S1.** Mendelian randomization (MR) association estimates for exposures on outcomes. **Table S2.** Sensitive analysis. **Table S3.** Multivariable Mendelian randomization (MR) association estimates of female traits and BMI, insomnia, FBG, hypertension across PhenoAge. **Table S4.** Multivariable Mendelian randomization (MR) association estimates of female traits and BMI, insomnia, FBG, hypertension across GrimAge. **Table S5.** Multivariable Mendelian randomization (MR) association estimates of female traits and BMI, insomnia, FBG, hypertension across HannumAge. **Table S6.** Multivariable Mendelian randomization (MR) association estimates of female traits and BMI, insomnia, FBG, hypertension across HorvothAge. **Table S7.** Genetic instruments for AFS. **Table S8.** Genetic instruments for AFB. **Table S9.** Genetic instruments for AAM. **Table S10.** Genetic instruments for ANM. **Table S11.** Genetic instruments for NSP. **Table S12.** Genetic instruments for insomnia. **Table S13.** Genetic instruments for hypertension. **Table S14.** Genetic instruments for bmi. **Table S15.** Genetic instruments for fasting blood glucose. **Table S16.** Characteristics of the GWAS use in exposures and confounders.

## Data Availability

UK Biobank data used in this work can be accessed at (https://www.ukbiobank.ac.uk). Summary statistics used in the analysis can be openly accessed at the IEU OpenGWAS project (https://gwas.mrcieu.ac.uk/). The original contributions presented in the study are included in the article, further inquiries can be directed to the corresponding author.
